# The prognostic significance of UCA1 for predicting clinical outcome in patients with digestive system malignancies

**DOI:** 10.18632/oncotarget.16534

**Published:** 2017-03-23

**Authors:** Fang-Teng Liu, Qing Dong, Hui Gao, Zheng-Ming Zhu

**Affiliations:** ^1^ Department of General Surgery, The Second Affiliated Hospital of Nanchang University, Nanchang 330000, Jiangxi Province, P.R. China; ^2^ Medical School of Nanchang University, Nanchang 330000, Jiangxi Province, P.R. China; ^3^ The Third Radiotherapy Department, Tumor Hospital of Jiangxi Province, Nanchang 330029, Jiangxi Province, P.R. China; ^4^ The Children's Hospital of Zhejiang University School of Medicine, Hangzhou 310052, Zhejiang Province, P.R. China

**Keywords:** UCA1, lncRNA, carcinoma, digestive system, prognosis

## Abstract

**Background:**

Urothelial Carcinoma Associated 1 (UCA1) was an originally identified lncRNA in bladder cancer. Previous studies have reported that UCA1 played a significant role in various types of cancer. This study aimed to clarify the prognostic value of UCA1 in digestive system cancers.

**Results:**

The meta-analysis of 15 studies were included, comprising 1441 patients with digestive system cancers. The pooled results of 14 studies indicated that high expression of UCA1 was significantly associated with poorer OS in patients with digestive system cancers (HR: 1.89, 95 % CI: 1.52–2.26). In addition, UCA1 could be as an independent prognostic factor for predicting OS of patients (HR: 1.85, 95 % CI: 1.45–2.25). The pooled results of 3 studies indicated a significant association between UCA1 and DFS in patients with digestive system cancers (HR = 2.50; 95 % CI = 1.30–3.69). Statistical significance was also observed in subgroup meta-analysis. Furthermore, the clinicopathological values of UCA1 were discussed in esophageal cancer, colorectal cancer and pancreatic cancer.

**Materials and methods:**

A comprehensive retrieval was performed to search studies evaluating the prognostic value of UCA1 in digestive system cancers. Many databases were involved, including PubMed, Web of Science, Embase and Chinese National Knowledge Infrastructure and Wanfang database. Quantitative meta-analysis was performed with standard statistical methods and the prognostic significance of UCA1 in digestive system cancers was qualified.

**Conclusions:**

Elevated level of UCA1 indicated the poor clinical outcome for patients with digestive system cancers. It may serve as a new biomarker related to prognosis in digestive system cancers.

## INTRODUCTION

Cancer is now becoming the leading cause of death in both developed and developing countries [1]. Digestive system malignant tumors occupy most of the all-cancer incidence and mortality, with 3.4 million new diagnosed cases and 1.5 million deaths each year [2]. The prognosis of patients with digestive system malignancies were unfavorable. Effective and accessible clinical biomarkers were urgently required for the prognosis prediction of patients with digestive system malignant tumors, since there was still no specific and accepted biomarker for this kind tumors.

LncRNAs were defined as non-protein coding RNAs with the length of more than 200 nucleotides. LncRNAs account for more than 80% of the entire genome transcripts [3]. Over the past decades, lncRNAs were always considered as transcriptions of “noise” or clonal artifacts [4]. Nowadays, more and more evidences showed that lncRNAs were closely associated with diverse biological processes [5–6]. Noteworthily, lncRNAs could be acted as oncogenes or tumor suppressors in cancers, such as HOTAIR, H19, MEG3 and TUSC7 [7–11].

Urothelial carcinoma associated 1 (UCA1) was a novel lncRNA gene with three exons and two introns, which is located in the chromosome 19p13.12. The lncRNA UCA1 was firstly discovered to be up-expressed in the carcinogenesis of bladder cancer in 2006 [12]. In recent years, increasing studies have reported that UCA1 was over-expressed in various cancers and UCA1 played important roles in the occurrence and development of human cancers [13–15]. In addition, many studies suggested that the expression of UCA1 was also related to prognosis of digestive system carcinomas [16–18]. These findings implied that UCA1 could be exploited as a potential biomarker for digestive system malignancies. However, until now, no specific meta-analysis was reported for assessing the prognostic value of UCA1 in digestive system carcinomas. Therefore, we performed this current comprehensive meta-analysis to evaluate the association between UCA1 expression level and prognosis in patients with digestive system cancers. The clinicopathological value of UCA1 was further analyzed in esophageal carcinoma, colorectal carcinoma and pancreatic carcinoma.

## RESULTS

### Literature search and study characteristics

**Figure 1:**
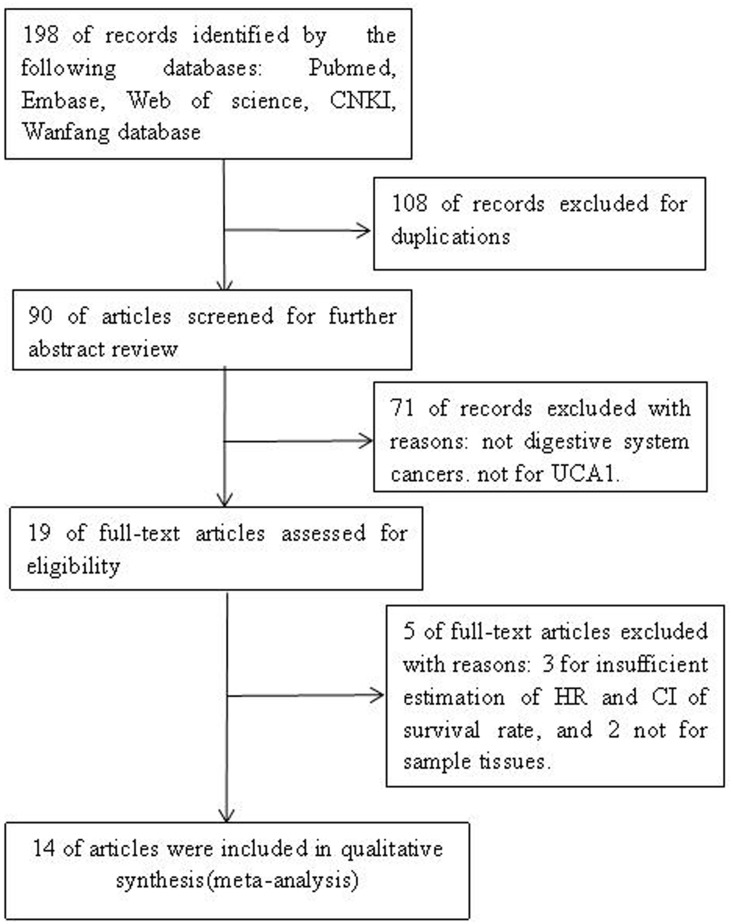
The steps for screening eligible publications

According to the criteria for selection mentioned above, after carefully screening the abstract and full-text of these references, finally, 14 publications (including 15 studies) [16–29] were identified as eligible for the present quantitative analysis of the prognostic value of UCA1 in digestive system cancers. All included publications were written in English. The detailed selection steps were shown (Figure 1).

A total of 1441 patients with survival data were included in this meta-analysis, the mean sample size was 96.07, with a maximum number of 240 and a minimum sample size of 20. Among the fifteen studies, six studies were about colorectal cancer [16, 19, 21, 24, 27], three studies were about gastric cancer [22, 25], two studies were about pancreatic cancer [26, 28], two studies were about hepatocellular carcinoma [20, 23], two studies were about esophageal carcinoma [17, 29]. The cancer cases in the included studies were all Asians (14 for Chinese, 1 for Koreans). The accrual period of 15 studies was ranged from 2014 to 2016. The participants in all the studies were categorized into high UCA1 expression group and low UCA1 expression group. The main characteristics were summarized (Table 1).

**Table 1:** Main characteristics of all studies included in the meta-analysis

First author, Year	Country	Cancer type	Sample Size	Tumor stage	Follow-up (months)	AT	Cut-off value	Detection method	Survival	Multivariate analysis	NOS score
Han Y, 2014 [16]	China	CRC	80	I-IV	Mean 42.6	NA	mean expression	qRT-PCR	OS	no	7
Li JY, 2014 [17]	China	EC	90	I-IV	median 43	None	mean expression	qRT-PCR	OS	yes	8
Gao JF, 2015 [18]	China	GC	20	I-IV	NA	None	NA	qRT-PCR	OS	yes	6
Tao K, 2015 [19]	China	CRC	80	I-IV	Over 60	None	the fourth quartile	qRT-PCR	OS	yes	8
Wang F, 2015 [20]	China	HCC	98	I-IV	Over 60	None	median expression	qRT-PCR	OS	yes	8
Ni BB, 2015 [21]	China	CRC	54	I-IV	Over 50	NA	median expression	qRT-PCR	OS	yes	7
Zheng Q, 2015 [22]	China	GC	112	I-IV	Over 60	None	median expression	qRT-PCR	OS,DFS	yes	8
Yang Z, 2015 [23]	Korea	HCC	240	I-IV	Over 60	None	median expression	NA	OS,DFS	OS-no.DFS-yes	8
Bian ZH-1, 2016 [24]	China	CRC	90	I-IV	Over 60	NA	median expression	qRT-PCR	OS	yes	7
Bian ZH-2, 2016 [24]	China	CRC	105	I-IV	Over 60	NA	median expression	qRT-PCR	OS	no	7
Shang C, 2016 [25]	China	GC	77	Borrmann type I-IV	Over 60	None	NA	qRT-PCR	DFS	yes	8
Chen P, 2016 [26]	China	PC	128	I-IV	1-60	None	mean expression	qRT-PCR	OS	yes	9
Jiang H, 2016 [27]	China	CRC	121	I-IV	1-60	None	median expression	qRT-PCR	OS	yes	8
Fu XL, 2016 [28]	China	PC	80	I-IV	Over 40	None	median expression	qRT-PCR	OS	yes	8
Jiao C, 2016 [29]	China	EC	66	I–III	1-30	NA	median expression	qRT-PCR	OS	no	6

CRC: colorectal cancer; EC: esophageal carcinoma; PC: pancreatic cancer; HCC: hepatocellular carcinoma; GC: gastric cancer; OS: overall survival; DFS: disease-free survival; qRT-PCR: quantitative real-time-polymerase chain reaction; AT: adjuvant therapy before surgery; NA: not available.

**Table 2:** Results of subgroup analysis of pooled hazard ratios of overall survival of patients with high UCA1 expression level

Stratified analysis	No. of studies	No. of patients	Pooled HR (95% CI)	p-value	Heterogeneity		
					I^2^ (%)	P-value	Model
[1] Tumor type	14	1364					
Colorectal cancer	6	530	2.21(1.35–3.08)	<0.001	0.0	0.989	Fixed effects
Pancreatic cancer	2	208	1.58(1.01–2.15)	<0.001	0.0	0.532	Fixed effects
Hepatocellular carcinoma	2	338	1.89(0.96–2.82)	<0.001	0.0	0.910	Fixed effects
Gastric cancer	2	132	2.13(1.17–3.09)	<0.001	0.0	0.746	Fixed effects
Esophageal carcer	2	156	2.41(1.01–3.82)	0.001	0.0	0.807	Fixed effects
[2] Histology type							
Squamous cell carcinoma	1	90	2.63(1.42–5.87)	<0.001	-	-	-
Nonsquamous cell carcinoma	13	1274	1.87(1.49–2.25)	<0.001	0.0	0.994	Fixed effects
[3] Country							
China	13	1124	1.89(1.50–2.27)	<0.001	0.0	0.990	Fixed effects
Korea	1	240	1.99(0.84–4.78)	0.117	-	-	-
[4] Sample size							
≥ 100	5	706	1.67(1.15–2.20)	<0.001	0.0	0.874	Fixed effects
< 100	9	658	2.10(1.57–2.63)	<0.001	0.0	0.997	Fixed effects
[5] Analysis type							
multivariate	10	873	1.85(1.45–2.25)	<0.001	0.0	0.950	Fixed effects
Non-multivariate	4	491	2.12(1.14–3.09)	<0.001	0.0	0.997	Fixed effects
[6] Cut-off value							
median expression	9	1094	2.12(1.53–2.70)	<0.001	0.0	0.998	Fixed effects
mean expression	3	170	1.62(1.05–2.19)	<0.001	0.0	0.563	Fixed effects
others	2	100	2.01(1.09–2.93)	<0.001	0.0	0.988	Fixed effects

### Prognostic value of UCA1 in digestive system cancers

#### UCA1 expression and overall survival(OS) in digestive system cancers

**Figure 2:**
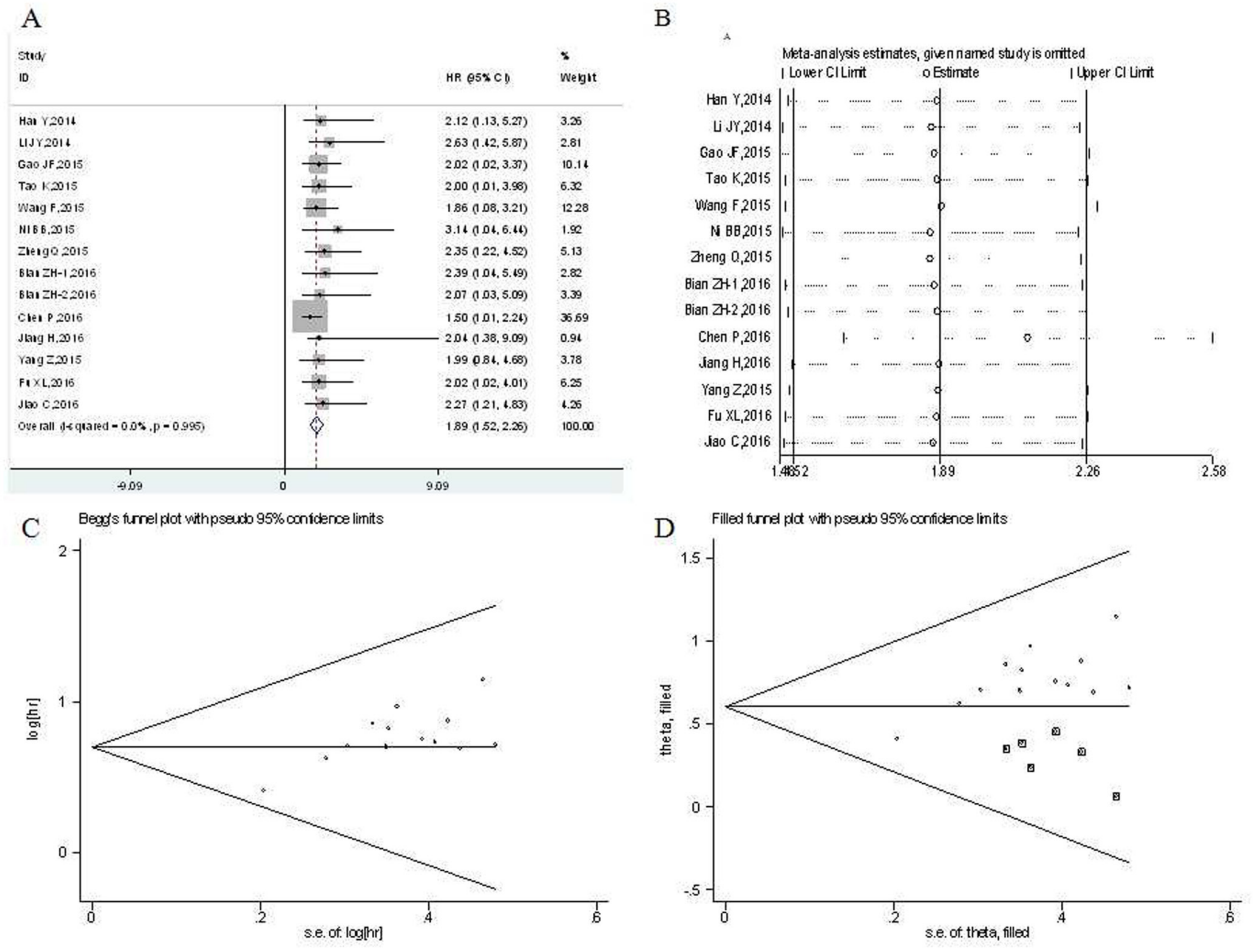
Meta-analysis of the pooled hazard ratios (HRs) of overall survival of patients with high UCA1 expression level in digestive system malignancies **(A)** Forest plot of HR for the relationship between increased UCA1 expression and OS. **(B)** Sensitivity analysis. **(C)** Begg's funnel plot. **(D)** Filled funnel plot of meta-analysis with “trim-and-fill” method. ○ indicated observed studies; ◘ indicated missed studies.

There was a total of 14 studies reporting the OS of 1364 patients per UCA1 expression levels [16–24, 26–29]. Because heterogeneity analysis showed no severe heterogeneity between studies, the fixed-effects model was applied in the meta-analysis (*P* = 0.995, I^2^ = 0.0 %). Overall, the pooled results confirmed that there was a significant association between high UCA1expression and poor OS in digestive system cancer (HR: 1.89, 95 % CI: 1.52–2.26, p<= 0.001) (Figure 2A).

Although there was no significant between-studies heterogeneity, the subgroup meta-analysis was conducted on the tumor type, country, histology type, analysis type, sample size and cut-off value (Table 1). From the subgroup results, we found that UCA1 was a significant prognostic indicator of OS for patients with esophageal cancer (HR: 2.41, 95 % CI: 1.01-3.82, p<= 0.001), gastric cancer (HR: 2.13, 95 % CI: 1.17-3.09, p<= 0.001), colorectal cancer (HR: 2.21, 95 % CI: 1.35–3.08, p<= 0.001), pancreatic cancer (HR: 1.58, 95 % CI: 1.01–2.15, p<= 0.001). A strong association was also showed in hepatocellular carcinoma (HR:1.89, 95 % CI: 0.96–2.82, p<= 0.001). Additionally, we detected a significant relationship between UCA1 expression and OS of patients with digestive system malignant tumors in China (HR: 1.89, 95 % CI: 1.50-2.27, p<= 0.001). UCA1 expression was found to be significantly associated with OS of patients with nonsquamous cell carcinoma (HR: 1.87, 95 % CI: 1.49–2.25, p<= 0.001). UCA1 was found to be significantly associated with OS of patients in studies reported in multivariate analysis (HR: 1.85, 95 % CI: 1.45–2.25, p<= 0.001) and non-multivariate analysis (HR: 2.12, 95 % CI: 1.14-3.09, p<= 0.001). The association between UCA1 and OS of patients was significant in studies with sample size both equal or greater than 100 (HR: 1.67, 95 % CI: 1.15-2.20, p<= 0.001) and less than 100 (HR: 2.10, 95 % CI: 1.57-2.63, p<= 0.001). Furthermore, a significant relationship between UCA1 and OS of patients was observed in both studies with the median value as cutoff (HR: 2.12 95 % CI: 1.53–2.70, p<= 0.001) and studies with the mean value as cutoff (HR: 1.62, 95 % CI: 1.05-2.19, p<= 0.001).

The sensitivity analysis was performed to assess the stability of the results by removing each study in turn. The result indicated that meta-analysis results didn't not change significantly (Figure 2B), which suggesting the robustness of the results. We assess the publication bias by the Begg's funnel plot and Egger's test. However, the shapes of funnel plot were asymmetric (Figure 2C), and publication bias was significant by Begg's test (z = 1.53, P = 0.125) and Egger's test (t[bias] = 4.51, P = 0.001). Nonparametric “trim-and-fill” method was used to replace six missing studies (Figure 2D). After the trim-and-fill adjustment, the estimated pooled HR was 1.823, with 95 % CI being 1.563–2.126 (p<= 0.001).

#### Independent prognostic value of UCA1 for OS in digestive system *cancers*

**Figure 3:**
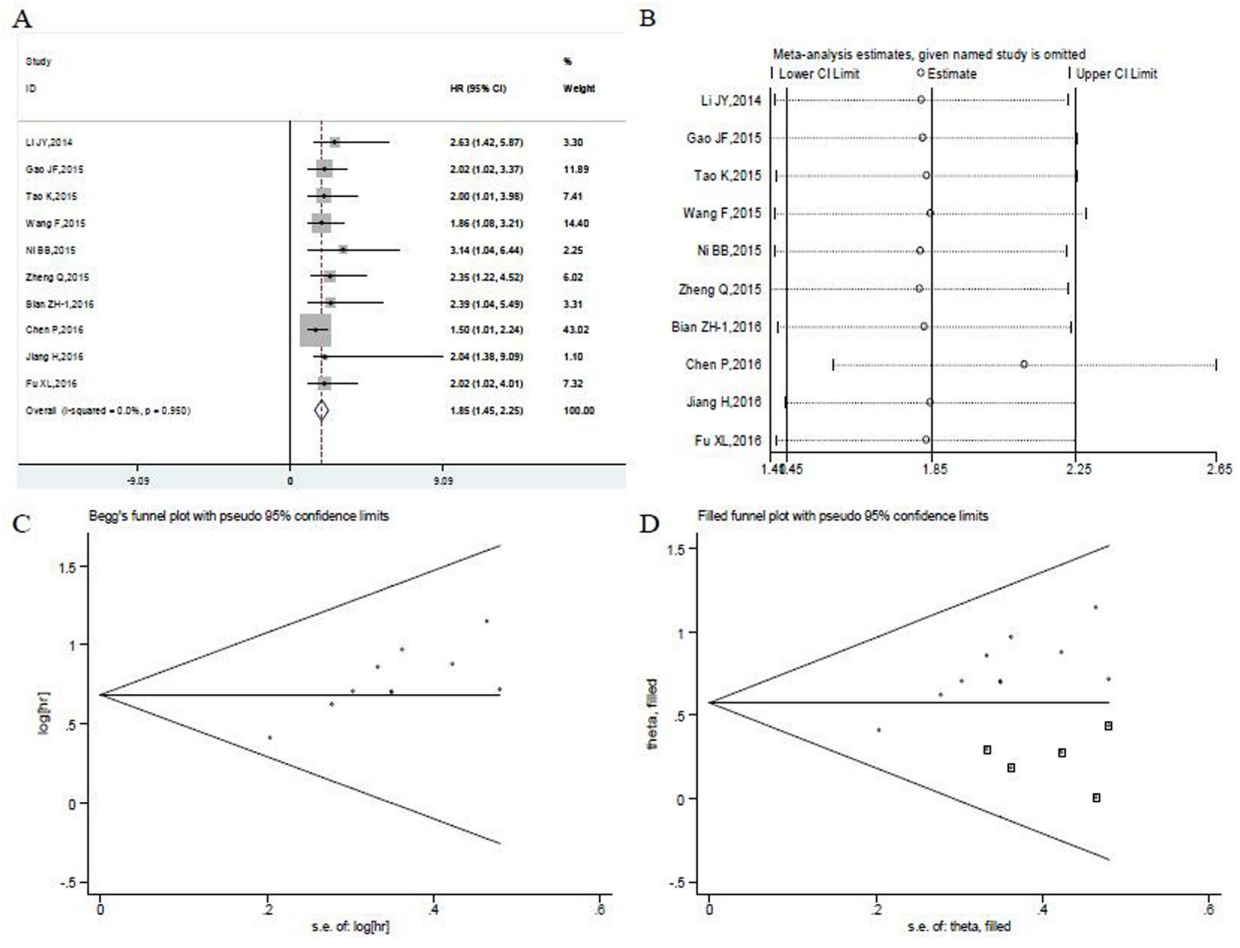
Meta-analysis of the independent predictive value of UCA1 for overall survival of patients with digestive system malignancies **(A)** Forest plots of meta-analysis. **(B)** Sensitivity analysis. **(C)** Begg's funnel plot. **(D)** Filled funnel plot of meta-analysis with “trim-and-fill” method. ○ indicated observed studies; ◘ indicated missed studies.

A total of 10 studies conducted the cox multivariate analysis to explore whether UCA1 was an independent predictive factor for OS of patients with digestive system malignancies [17–22, 24, 26–68]. There was no significant heterogeneity existing among studies (P= 0.950, I^2^= 0.0%), the fixed-effects model was utilized to combine the hazard ratios (HRs) with corresponding 95 % CI. The pooled meta-analysis confirmed that high UCA1 expression was a significant independent predictor of poor OS in digestive system malignancies (HR: 1.85, 95 % CI: 1.45–2.25, p<= 0.001) (Figure 3A). The patients detected with elevated UCA1 expression were more likely to have significantly shorter OS.

Moreover, the pooled HR values > 1 were consistently calculated in subgroup meta-analysis stratified by the tumor type, histology type, sample size and cut-off value, which still had statistical significance (Table 3). The subgroup analysis showed the above factors did not alter the predictive value of UCA1 as an independent factor for OS of patients with digestive system malignancies. And no obvious heterogeneity was observed in subgroup analysis of studies looking at the independent role of UCA1 (Table 3).

**Table 3:** Results of subgroup analysis of the independent role of UCA1 in overall survival of digestive system malignancies

Stratified analysis	No. of studies	No. of patients	Pooled HR(95% CI)	p-value	Heterogeneity		
					I^2^ (%)	P-value	Model
[1] Tumor type	10	873					
Colorectal cancer	4	345	2.28(1.20–3.36)	<0.001	0.0	0.909	Fixed effects
Pancreatic cancer	2	208	1.58(1.01–2.15)	<0.001	0.0	0.532	Fixed effects
Hepatocellular carcinoma	1	98	1.86(1.08–3.21)	<0.001	-	-	-
Gastric cancer	2	132	2.13(1.17–3.09)	<0.001	0.0	0.746	Fixed effects
Esophageal carcinoma	1	90	2.63(1.42–5.87)	<0.001	-	-	-
[2] Histology type							
Squamous cell carcinoma	1	90	2.63(1.42–5.87)	<0.001	-	-	-
Nonsquamous cell carcinoma	9	783	1.82(1.41–2.23)	<0.001	0.0	0.944	Fixed effects
[3] Sample size							
≥ 100	3	361	1.62(1.04–2.19)	<0.001	0.0	0.625	Fixed effects
< 100	7	512	2.09(1.51–2.66)	<0.001	0.0	0.982	Fixed effects
[4] Cut-off value							
median expression	6	603	2.12(1.43–2.81)	<0.001	0.0	0.968	Fixed effects
mean expression	2	170	1.58(0.99–2.18)	<0.001	0.0	0.340	Fixed effects
others	2	100	2.01(1.09–2.93)	<0.001	0.0	0.988	Fixed effects

The leave-one-out sensitivity analysis performed showed that no individual study changed the pooled HRs significantly (Figure 3B). However, the shape of funnel plot was asymmetrical (Figure 3C) and the tests of publication bias showed there was significant publication bias (Begg's test: z (continuity corrected) = 1.79, P=0.074; Egger's test: t(bias)=4.62, P = 0.002). Then the “trim and fill method” was also adopted to replace five missing studies(Figure 3D). After correction, the adjusted pooled HR was 1.775 (95 % CI: 1.491- 2.113, p<= 0.001).

#### UCA1 expression and disease-free survival(DFS) in digestive system cancers

**Figure 4:**
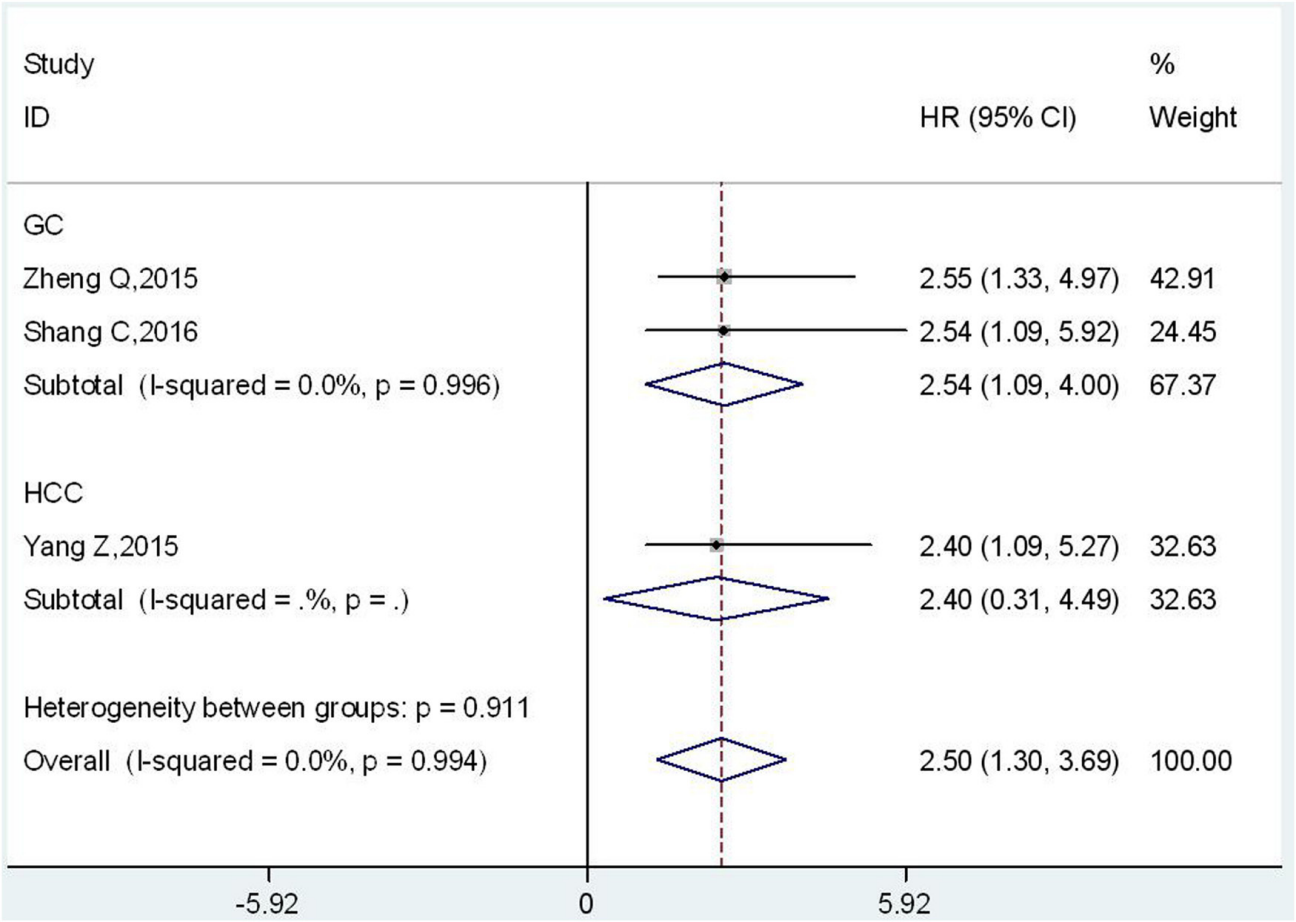
Forest plot of HR for the relationship between high UCA1 expression level and DFS in digestive system malignancies

Only three studies including 429 patients reported the relationship between UCA1 and DFS in digestive system malignancies [22, 23, 25]. Two studies were about gastric cancer [22, 25], and one was for hepatocellular carcinoma [23]. Due to no significant heterogeneity across-studies was observed (I^2^ = 0.0 %; P = 0.994), the fixed-effects model was used to analyze the pooled hazard ratios (HRs) with corresponding 95 % confidence interval (CI). The results showed that there was a significant association between high expression level of UCA1 and poor DFS in digestive system malignancies (HR = 2.50; 95 % CI = 1.30–3.69; p<= 0.001) (Figure 4). Particularly, UCA1 was found to be a significant prognostic indicator of DFS for patients with gastric cancer (HR =2.54; 95 % CI = 1.09–4.00, p= 0.001) (Figure 4). However, due to no heterogeneity across-studies and the small number of studies, publication bias and sensitivity analysis were not conducted.

### Clinicopathological value of UCA1 in digestive system cancers

#### UCA1 expression and clinicopathological factors in esophageal cancer

There were only two studies reporting the relationship between UCA1 and clinicopathological features of esophageal cancer [17, 29]. Both two studies assessed the correlation between UCA1 expression and gender, age and TNM stage. Because no significant heterogeneity was detected in gender (P= 0.97, I^2^ %= 0%), age (P=0.80, I^2^ %= 0%) and TNM stage (P=0.87, I^2^ %= 0%), the fixed-effects model was used for all (Table 4). The combined results showed that there was no significant association betweenUCA1expression and gender (OR: 1.26, 95 % CI: 0.67–2.38, p = 0.47) and age (OR: 1.35, 95 % CI: 0.69–2.65, p = 0.38). While high UCA1 expression was found to be significantly associated with advanced TNM stage (OR: 3.81, 95 % CI: 1.87–7.76, p= 0.0002). Publication bias and sensitivity analysis were not applicable in analyzing the relationship between UCA1 and clinicopathological features of esophageal cancer, because of the limited number of studies without between-studies heterogeneity.

**Table 4:** Results of meta-analysis of high UCA1 expression level and clinicopathological features in esophageal cancer, colorectal cancer and pancreatic cancer

Stratified analysis	No. of studies	No. of patients	Pooled OR (95% CI)	p-value	Heterogeneity		
					I^2^ (%)	P-value	Model
**Esophageal cancer**	2	156					
Gender (male vs. female)	2	156	1.26(0.67–2.38)	0.47	0.0	0.97	Fixed effects
Age (≥60 vs. <60)	2	156	1.35(0.69–2.65)	0.38	0.0	0.80	Fixed effects
Tumor stage (III+IV vs. I+II)	2	156	3.81(1.87–7.76)	0.0002	0.0	0.87	Fixed effects
**Colorectal cancer**	5	425					
Gender (male vs. female)	5	425	0.76(0.51–1.13)	0.17	16	0.31	Fixed effects
Tumor size (≥5 vs. <5cm)	3	250	1.81(0.67–4.90)	0.24	70	0.04	Random effects
Location (colon vs rectum)	3	291	0.77(0.48–1.24)	0.28	0.0	0.8	Fixed effects
Histological grade (poorly and others vs. well and moderately)	5	425	2.60(1.67-4.03)	<0.0001	45	0.12	Fixed effects
Depth of invasion (T3-4 vs. T1-2)	4	371	1.71(0.78–3.75)	0.18	60	0.04	Random effects
Lymphatic invasion (yes vs. no)	3	224	1.56(0.88–2.75)	0.13	0.0	0.73	Fixed effects
Venous invasion (yes vs. no)	2	134	0.85(0.39–1.85)	0.69	0.0	0.78	Fixed effects
Lymph node metastasis (yes vs. no)	2	134	3.88(1.71–8.83)	0.001	0.0	0.74	Fixed effects
Distant metastasis (yes vs. no)	4	345	2.67(1.32–5.38)	0.006	25	0.26	Fixed effects
Tumor stage (III+IV vs. I+II)	5	425	2.45(1.62–3.70)	<0.0001	38	0.17	Fixed effects
**Pancreatic cancer**	2	208					
Gender (male vs. female)	2	208	0.92(0.53–1.61)	0.78	33	0.22	Fixed effects
Location (head vs. body and tail)	2	208	1.14(0.64–2.02)	0.66	0.0	0.52	Fixed effects
Histological grade (poorly vs. well and moderately)	2	208	1.04(0.39–2.79)	0.94	67	0.04	Random effects
Depth of invasion (T3-4 vs. T1-2)	2	208	2.50(1.34–4.67)	0.004	0.0	0.41	Fixed effects
Nervous invasion (yes vs. no)	2	208	1.51(0.86–2.66)	0.15	0.0	0.36	Fixed effects

#### UCA1 expression and clinicopathological factors in colorectal cancer

A total of five studies explored the relationship between UCA1 and clinicopathological characteristics of colorectal cancer [16, 19, 21, 24, 27], including five studies regarding gender, differentiation grade and TNM stage, four studies of depth of primary tumor invasion and distant metastasis, three studies involving tumor size, location and lymphatic invasion, two studies of lymph node metastasis and venous invasion (Table 4).

The analysis between UCA1 expression and gender (P = 0.31, I^2^ %= 16 %), location (P = 0.80, I^2^ %= 0 %), differentiation grade (P = 0.12, I^2^ %= 45 %), lymphatic invasion(P = 0.73, I^2^ %= 0 %), venous invasion (P = 0.78, I^2^ %= 0 %), lymph node metastasis (P = 0.74, I^2^ %= 0 %), distant metastasis (P = 0.26, I^2^ %= 25 %), and TNM stage (P = 0.17, I^2^ %= 38 %) in colorectal cancer revealed no significant heterogeneity across studies, and thus the fixed-effects model was applied. However, there were significant heterogeneity in studies regarding depth of primary tumor invasion (P = 0.04, I^2^ %= 60 %) and tumor size (P = 0.04, I^2^ %= 70 %) (Table 4), therefore the random-effects model was applied.

In colorectal cancer, high UCA1 expression was significantly related to differentiation grade (OR: 2.60, 95 % CI: 1.67– 4.03, p<0.0001), lymph node metastasis (OR: 3.88, 95 % CI: 1.71– 8.83, p = 0.001) and distant metastasis(OR: 2.67, 95 % CI: 1.32-5.38, p = 0.006) and TNM stage (OR: 2.45, 95 % CI: 1.62-3.70, p <0.0001). Whereas no significant association was found with gender (OR: 0.76, 95 % CI: 0.51– 1.13, p = 0.17), tumor size (OR: 1.81, 95 % CI: 0.67– 4.90, p = 0.24), location (OR: 0.77, 95 % CI: 0.48– 1.24, p = 0.28), the depth of tumor(OR: 1.71, 95 % CI: 0.78– 3.75, p = 0.18), lymphatic invasion (OR: 1.56, 95 % CI: 0.88-2.75, p = 0.13) and venous invasion (OR: 0.85, 95 % CI: 0.39-1.85, p = 0.69). Publication bias and sensitivity analysis were not performed due to the small number of studies and little heterogeneity.

#### UCA1 expression and clinicopathological factors in pancreatic cancer

In pancreatic cancer, there were only two studies assessing the correlation between UCA1 expression and clinicopathological data [26, 28], the following clinicopathological features were both reported in the two studies: gender, location, histological grade, nervous invasion and depth of tumor.

Except for histological grade (P = 0.04, I^2^%= 67%), there was no significant heterogeneity between studies in gender (P = 0.22, I^2^ %= 33 %), location (P = 0.52, I^2^%= 0 %), nervous invasion (P =0.36, I^2^ %= 0 %) and depth of invasion (P = 0.41, I^2^ %= 0 %). Thus, the fixed-effects model was used for all except for the histological grade (Table 4).

The overall meta-analysis results revealed that UCA1 expression was no significantly associated with gender (OR: 0.92, 95 % CI: 0.53–1.61, p = 0.78), location (OR:1.14, 95 % CI: 0.64–2.02, p = 0.66), histological grade (OR: 1.04, 95 % CI: 0.39–2.79, p = 0.94) and nervous invasion (OR: 1.51, 95 % CI: 0.86–2.66, p = 0.15) (Table 4). Nevertheless, the depth of primary tumor invasion has a significant correlation with UCA1 expression (OR: 2.50, 95 % CI: 1.34–4.67, p = 0.004) (Table 4).

Because of little heterogeneity and small number of included studies, publication bias and sensitivity analysis were not performed in analyzing the relationship between UCA1 and clinicopathological parameters of pancreatic cancer.

## DISCUSSION

Recently, many studies reported that the expression of UCA1 was significantly upregulated in tumor tissues from human digestive system, and UCA1 played a crucial role in the tumorigenesis of digestive system malignancies [30–32]. But the prognostic value of UCA1 in the malignant digestive system tumors was still not clear. Therefore, this meta-analysis was performed to evaluate the clinical relevance and prognostic value of UCA1 in digestive system malignancies. To our best knowledge, this is the first meta-analysis to explore the association between UCA1 expression and OS, DFS and clinicopathological features in human digestive system cancers.

Here we undertook meta-analysis of 15 studies comprising 1441 patients with digestive system cancer. 1364 patients with OS data were included in 14 studies and 429 patients with DFS data were from 3 studies. The prognostic value of UCA1 in digestive system cancers was assessed. However, both the heterogeneity test and fixed-effects model were performed. The present study demonstrated that UCA1 expression was negatively correlated with OS in patients with digestive system malignancies (HR: 1.89, 95 % CI: 1.52–2.26, p<= 0.001). The patients with high UCA1 expression had a poorer OS compared to patients with low UCA1 expression. Despite significant heterogeneity didn't exist in these studies for OS, subgroup analyses were performed on the tumor type, country, histology type, analysis type, sample size and cut-off value (Table 2), these factors didn't alter the significant predictive value of UCA1 in OS in different kinds of digestive system malignancies. Then the “trim-and-fill” method was applied to adjust our results since we found significant publication bias through Begg's test and Egger's test in the studies (Figure 2). After adjusting for the pooled HR and 95 % CI, there was no significant alternation with the primary data (HR: 1.823, 95 % CI: 1.563–2.126, p<= 0.001). It indicated that our results were reliable, which was also confirmed in sensitivity analysis.

Furthermore, we found that UCA1 could act as an independent prognostic prediction factor for patients with digestive system malignancies. The results could be confirmed by HRs and 95 % CIs from multivariate Cox regression analyses (HR:1.85, 95 % CI: 1.45-2.25, p<= 0.001). And no obvious heterogeneity was observed in subgroup analysis of studies. The prognostic implication of UCA1 was also showed in the stratified analysis based on cancer type, histology type, sample size and cut-off value (Table 3). None of these variables changed the predictive value of UCA1 as an independent factor for OS of patients with digestive system malignancies. Although significant publication bias was found in our *meta*-*analysis*, the adjusted pooled HR demonstrated that UCA1 could be applied as an independent prognostic factor for digestive system malignancies (HR: 1.775, 95 % CI: 1.491- 2.113, p<= 0.001) (Figure 3). The sensitivity analysis also indicated the stability of this results (Figure 3). Besides that, our results also showed that there was a significant negative association between UCA1 levels and DFS in digestive system malignancies (HR = 2.50; 95 % CI = 1.30–3.69; p<= 0.001) (Figure 4), particularly in gastric cancer (HR =2.54; 95 % CI = 1.09–4.00, p= 0.001) (Figure 4).

For the relationship between UCA1 and clinicopathological features in digestive system cancers, multiple studies have been performed on different kinds of the cancers. But clinicopathological value of UCA1 in a specific kind of digestive system cancer was distinctive and even contradictory. For example, in human colorectal cancer, Han *et al*. [16] suggested that UCA1 correlated with differentiation and invasion depth. Jiang *et al*. [27]indicated that UCA1 was correlated with differentiated histology, but no significant association was found in invasion depth of colorectal cancer. Chen *et al*. [26] reported different result, which demonstrated that UCA1 expression in pancreatic cancer was significantly correlated with depth of invasion, but without no significant correlation with histological differentiation.

The molecular biology characters were different and mechanism of tumorigenesis was complex in the digestive tract tumor. Thus, we focused on the clinicopathological value of UCA1 in a specific type of digestive tract cancers. We studied the association between UCA1 expression and clinicopathological characteristics in esophageal cancer, colorectal cancer and pancreatic cancer. The clinicopathological value of UCA1 in gastric cancer and hepatocellular carcinoma was not obtained for the limited and unavailable data of clinical pathological features. In esophageal cancer, we found UCA1 was significantly associated with advanced TNM stage, but no significant association was found in gender and age. In colorectal cancer, our research demonstrated that UCA1 expression was significantly related to differentiation grade, lymph node metastasis, distant metastasis and TNM stage, but no significant association was observed in gender, tumor size, location, the depth of tumor, lymphatic and venous invasion. Finally, the clinicopathological role of UCA1 was evaluated in pancreatic cancer, UCA1 expression was significantly associated with primary tumor invasion, however no significant association was found in gender, location, histological grade and nervous invasion.

The expression patterns and biological roles of UCA1 in digestive system cancers was explored. It would further support UCA1 as a promising biomarker for the prognosis of digestive system tumors. The potential roles of UCA1 expression regulation in various digestive carcinomas have been reviewed. For gastric cancer, UCA1 was observed to be highly expressed, while the silence of UCA1 could decrease proliferation of tumor cells. Expression of UCA1 was negatively correlated with the miR-27b and the UCA1-miR-27b axis was involved in regulation of chemo-sensitivity of gastric cancer cells [33]. In esophageal squamous cell carcinoma (ESCC), it demonstrated that UCA1 could inhibited cell proliferation, migration, invasion, and cell cycle progression of EC109 cells, and UCA1 could involve in ESCC development by regulating the Wnt signaling pathway [34]. For colorectal cancer (CRC), Han et al. [16] found that UCA1 levels were markedly elevated in tissues and cells compared to controls, and it could influence malignant biological behavior of CRC cells. The study by Bian *et al*. [24] identified UCA1 as a new oncogene, which implicated in miR-204-5p-CREB1/BCL2/RAB22A regulatory network in CRC. For the liver cancer, it found that upregulated UCA1 could promote cell growth and tumorigenesis through the signaling of HBx-UCA1/EZH2-p27Kip1 axis in hepatocarcinogenesis [35]. For the contribution of UCA1 to tumorigenesis of pancreatic cancer, Chen *et al*. [26] detected UCA1 expression was greatly increased in cancerous tissue and UCA1 played a physiological role in regulating proliferation, apoptosis and cell cycle arrest. Fu *et al*. [28] also provide evidence that UCA1 promoted the tumorigenesis in pancreatic cancer. Nevertheless, the exact molecular mechanisms of UCA1 in carcinogenesis and progression of digestive system cancers was still pending exploration. More experimental studies should be conducted to clarify the unrecognized roles and detailed function of UCA1 in carcinogenesis and progression.

It should be emphasized that the present meta-analysis had several limitations. First, the criterion of high expression for UCA1 in tissue samples was not the same in different studies, and it was hard to get a consensus cut-off value to define the UCA1 overexpression in various cancers. Second, the therapeutic strategy was diverse in gastrointestinal tumors, which made a significant influence on postoperative survival of patients, leading to heterogeneity. Third, we only studied publications written in English and Chinese, and only 15 studies with 1441 patients were included in present meta-analysis, so the total number of studies and patients included was relatively small. Fourth, only 3 studies including 429 patients were included and reported the relationship between UCA1 and DFS. Furthermore, all the studies we included were conducted in Asian population from Chinese and Korea. Finally, the potential publication bias was also observed in this meta-analysis and positive results would be more easily to be published than that of in negative results.

In conclusion, the meta-analysis results of this study could help to improve our understanding on the prognosis significance of UCA1 in different types of digestive system carcinomas. UCA1 may serve as a novel biomarker for predicting the prognosis and assessing clinicopathologic features in digestive system carcinomas. Ultimately, further scientific research with larger-size, multi-center and higher-quality studies is required to verify the clinical utility of UCA1 in digestive system malignancies.

## MATERIALS AND METHODS

### Search strategy and study selection

To get access to potentially eligible studies, comprehensive literature retrieval was performed against several databases: PubMed, Web of Science, Embase and Chinese National Knowledge Infrastructure (CNKI), Wanfang database. The literature search was conducted up to Oct. 15, 2016. The publications were identified with the combination of the following search terms: “Urothelial cancer-associated 1” or “UCA1” or “lncRNA UCA1” or “long noncoding RNA UCA1”, “cancer” or “carcinoma” or “tumor” or “neoplasm,” “prognosis” or “survival” or “clinical outcome”. The reference lists of relevant articles were also searched manually. The published language was limited to English and Chinese.

Inclusion and exclusion criteria were set to screen the publications. Inclusion criteria were as follows: (1) The studies explored the association between UCA1 and cancer prognosis; (2) Related clinicopathologic parameters were described; (3) The UCA1 expression were determined in the tissues of digestive system cancers; (4) Patients were divided into high and low expression groups according to the expression level of lncRNA UCA1; (5) Sufficient information and data was provided for calculating a hazard ratio (HR) with its 95% confidence interval (CI). Exclusion criteria were as follows: (1) The studies not relevant to digestive system cancers, UCA1, or cancer prognosis; (2) studies without usable data, such the data was obtained from animal models; (3) duplicate publications; and (4) reviews, letters and case reports.

### Data extraction and quality assessment

Two authors (DQ and GH) extracted data independently from all the including studies by standardized data compilation forms, and any disagreements were resolved by discussion. For all eligible studies, the following information was collected: the first author, year of publication, country, cancer type, sample size, tumor stage, follow-up period, outcome measures, cut-off value, determination method, HR and corresponding 95 % CI. The clinicopathological data were also extracted from the eligible studies.

If the HRs with corresponding 95 % CIs was provided in a including study, the available data was directly extracted. If a study provided only Kaplan-Meier curves, the survival data was extracted from Kaplan-Meier survival curves by Engauge Digitizer V4.1 (http://digitizer.sourceforge.net/).

The Newcastle-Ottawa Scale (NOS) was applied to assess the quality of all including studies. The scores of NOS criteria were ranged from 0 (lowest) to 9 (highest). If the final scores of a study was higher, the methodological quality was better. A study with an NOS score equal or more than 6 was considered to be of high quality. In this meta-analysis, the quality of all studies included in this meta-analysis was varied from 6 to 9, with a mean value of 7.5.

### Statistical analysis

Statistical analyses of HRs for OS and DFS were calculated by Stata SE12.0 (*Stata Corporation, College Station, TX, USA*). ORs for clinicopathological parameters were calculated by RevMan5.3 software *(Cochrane Collaboration*, http://community.cochrane.org/tools/reviewproduction-tools/revman-5/revman-5-download).

The between-studies heterogeneity was determined by Chi square-based Q test and I^2^ statistics. A P value great than 0.05 for the Q test and I^2^ value less than 50 % were considered to be of no significant heterogeneity, then the fixed effects model was applied (P > 0.05, I^2^ < 50%); otherwise, the random effects model was used to provide wider CI for studies with significant heterogeneity.

Subgroup analysis was performed to further investigate the prognostic value of lncRNA UCA1 in digestive system malignancies. Potential publication bias was determined with funnel plots analysis, Begg's test and Egger's test. The sensitivity analysis was also performed to assess the stability of the results. A p value less than 0.05 was statistically significant.
